# Leveraging Machine Learning for Optimized Mechanical Properties and 3D Printing of PLA/cHAP for Bone Implant

**DOI:** 10.3390/biomimetics9100587

**Published:** 2024-09-27

**Authors:** Francis T. Omigbodun, Norman Osa-Uwagboe, Amadi Gabriel Udu, Bankole I. Oladapo

**Affiliations:** 1 Wolfson School of Mechanical, Electrical and Manufacturing Engineering, Loughborough University, Loughborough LE11 3TU, UK; f.omigbodun@lboro.ac.uk (F.T.O.); n.osa-uwagboe@lboro.ac.uk (N.O.-U.); 2 The Manufacturing Technology Centre, Coventry CV7 9JU, UK; 3 Air Force Research and Development Centre, Nigerian Air Force Base, Kaduna PMB 2104, Nigeria; 4 School of Engineering, University of Leicester, Leicester LE1 7RH, UK; 5 School of Science and Engineering, University of Dundee, Dundee DD1 4HN, UK

**Keywords:** machine learning, predictive modelling, data-driven optimization, regression algorithms, artificial intelligence in biomedical engineering, additive manufacturing

## Abstract

This study explores the fabrication and characterisation of 3D-printed polylactic acid (PLA) scaffolds reinforced with calcium hydroxyapatite (cHAP) for bone tissue engineering applications. By varying the cHAP content, we aimed to enhance PLA scaffolds’ mechanical and thermal properties, making them suitable for load-bearing biomedical applications. The results indicate that increasing cHAP content improves the tensile and compressive strength of the scaffolds, although it also increases brittleness. Notably, incorporating cHAP at 7.5% and 10% significantly enhances thermal stability and mechanical performance, with properties comparable to or exceeding those of human cancellous bone. Furthermore, this study integrates machine learning techniques to predict the mechanical properties of these composites, employing algorithms such as XGBoost and AdaBoost. The models demonstrated high predictive accuracy, with R^2^ scores of 0.9173 and 0.8772 for compressive and tensile strength, respectively. These findings highlight the potential of using data-driven approaches to optimise material properties autonomously, offering significant implications for developing custom-tailored scaffolds in bone tissue engineering and regenerative medicine. The study underscores the promise of PLA/cHAP composites as viable candidates for advanced biomedical applications, particularly in creating patient-specific implants with improved mechanical and thermal characteristics.

## 1. Introduction

Tissue engineering is a rapidly developing multidisciplinary discipline at the crossroads of biology, engineering, and materials science that has already produced revolutionary advances in regenerative medicine and other biomedical fields [1,2,3]. Creating biocompatible scaffolds that can imitate the extracellular matrix, provide structural support, and direct the regeneration of tissues and organs is crucial to advancing tissue engineering [4,5]. Recent advances include the development of bioinks that can be used for 3D bioprinting living tissues, using stem cells to regenerate damaged tissues, and the creation of scaffolds that can release growth factors to aid tissue healing and regeneration. These advancements have significantly pushed the boundaries of what can be achieved in tissue repair and reconstruction. Hence, novel engineering techniques, such as additive manufacturing, have gained traction recently, partly due to traditional methods being costly, time-consuming, and challenging in terms of customizability and scalability. Additive manufacturing, specifically fused deposition modelling (FDM) 3D printing, has efficiently and accurately manufactured patient-specific implants and complicated tissue constructions [6,7,8,9,10]. Because of its biocompatibility, bioresorbability, and processability, PLA has become a polymer of interest in tissue engineering [11,12]. PLA is a biodegradable polymer made from renewable resources [13,14,15]. Researchers have been looking for novel ways to improve PLA’s mechanical qualities without compromising its biocompatibility because of its mechanical limitations, such as its comparatively low strength and stiffness compared to natural tissues [16,17]. At the same time, introducing reinforcing materials such as cHAP, a bioactive ceramic with a chemical composition similar to natural bone mineral, has emerged as a viable method to solve the mechanical inadequacies of PLA scaffolds [18]. cHAP has been widely studied for its osteoconductive properties, and its incorporation into PLA scaffolds has been shown to improve mechanical strength and bioactivity significantly, making the composites more suitable for load-bearing applications in bone repair and regeneration [19,20,21]. Artificial intelligence (AI) can be employed in predicting various composite mechanical properties, resulting in cost and time savings on experimentation while mitigating the intricacies of ascertaining precise volumes of additives [10,22,23]. For instance, in [24], a predictive model for the biomechanical properties of AM polydopamine (PDM)-coated PLA bone plates using various ML algorithms was developed. Their study suggested that long short-term memory was the best model for predicting the tensile and flexural strength of PDM-coated PLA bone plates. Alakent et al. [24] predicted the tensile strength of PLA-based composites based on synthesis features, including chemical composition, molecular weight, manufacturing method, compounding and testing temperature, and filler types employed [25,26]. Their results exposed the role of temperature and other synthesis features in determining the tensile strength for varying manufacturing methods.

Similarly, Thakur et al. [25] utilised an ML classification and regression trees method was used to model peak strength and strength at break [27]. Their study revealed that a combination of parameters, including the 0° orientation of carbon fibre deposition, a nozzle temperature of 205 °C, and a bed temperature of 55 °C, represent the optimal settings for manufacturing PLA-carbon fibre composite structures. Munir et al. [26] researched real-time monitoring of extrusion-induced degradation in various grades of PLA under diverse process conditions and machine configurations [28]. They aimed to determine the most effective method for creating interpretable models with high predictive accuracy and robustness across different process settings. Their study compared the performance of a Recursive Feature Elimination (RFE) approach with conventional dimension reduction and regression techniques. The findings indicated that RFE-based soft sensors showed promise for enhancing quality control in PLA processing, enabling monitoring molecular weight degradation during processing across different machine settings. Although various works have studied the predictive performance of PLA mechanical properties, the effects and use of PLA with various cHAP composite volumes for bone tissue engineering are still unclear regarding their enhanced mechanical and thermal properties and their compatibility for medical applications. Therefore, a novel PLA/cHAP composite scaffold is proposed in this paper for biomedical applications to address these concerns.

**Figure 1:**
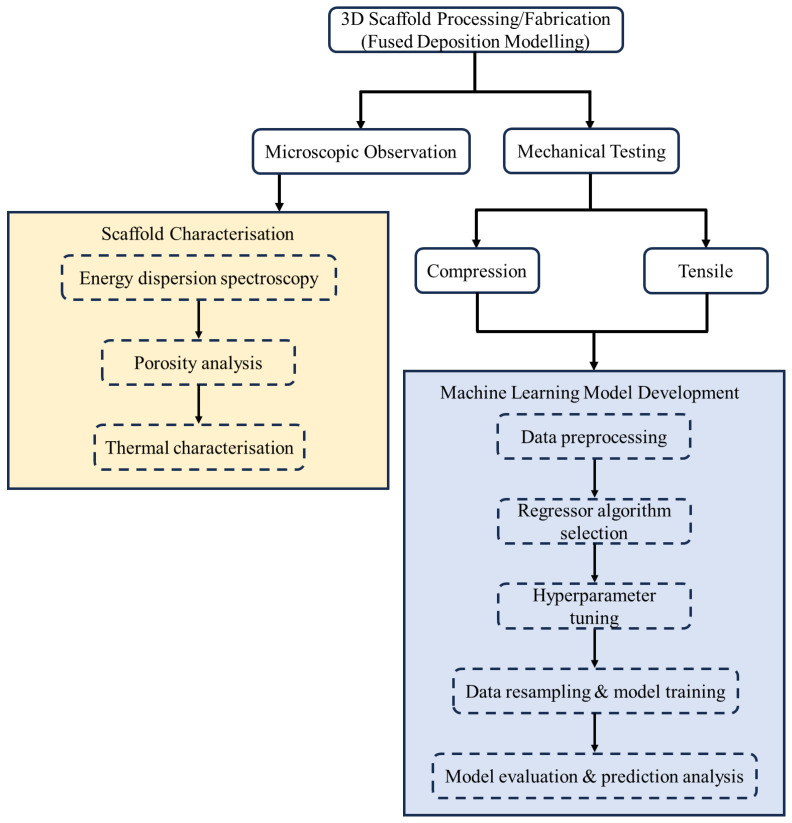
Research setup.

This research investigates the viability of using FDM 3D printing to create PLA/cHAP composite scaffolds with enhanced mechanical and thermal characteristics for use in various biomedical settings [29]. Mechanical performance, thermal stability, and biocompatibility of the resultant composite scaffolds are carefully studied as a function of cHAP concentration in the PLA matrix. The study establishes a predictive framework employing multiple machine learning regression algorithms. Models exhibiting superior performance were chosen based on metrics such as mean square error (MSE), mean absolute error (MAE), and coefficient of determination (R^2^). These selected models were then compared and validated for accuracy, robustness, and reliability in predicting the mechanical properties of the composite under study. The research focused on compressive and tensile strengths as response variables due to their crucial role in assessing the composite’s suitability for bone tissue engineering. The ability of PLA and varying amounts of cHAP to withstand mechanical stress directly impacts their potential as robust bone replacement materials. Therefore, employing this methodology to characterise these properties can aid in developing more efficient and durable PLA/cHAP composites for biomedical applications, particularly in bone tissue engineering and regeneration. This paper is organised as follows: Section 2 and Section 3 detail the experimental approaches adopted and the study results, respectively, as described in Figure 1. The research findings are highlighted in the Conclusion, and their implications for biomedical utilisation are discussed.

## 2. Materials and Methods

### 2.1. Materials

PLA purchased from Nature Works LLC, Plymouth, MN, USA, under the trade name of Ingeo™ Biopolymer 4060D, was used to fabricate the composite scaffolds. Table 1 shows the PLA data sheet from the manufacturer. PLA biopolymer with a molecular weight of 16g/mol, semicrystalline and transparent, allows it to be processed by conventional procedures such as extrusion, thermoforming, and injection [30,31]. The specifications of this product given by the manufacturer are presented in Table 2 below. The material used to reinforce the polymeric phase was non-sintered calcium hydroxyapatite from HIMED, Himteco Medical Applications, Inc., New Work, NY, USA. Calcium hydroxyapatite was selected to fully degrade with the polymeric matrix and promote the bioactivity of the material, analytical grade chloroform, Chloroform, HPLC, from the JT. The solvent casting technique used the Baker brand as a solvent for the manufacturing process of composite materials.

### 2.2. Methods

#### 2.2.1. Preparation of PLA-cHAP Composites

The composites were made in two categories to meet the study’s goals. Initially, PLA-cHAP composites had 0, 7.5, and 10% cHAP. The designation of the prepared nanocomposites is named in Table 2. In the following order, the PLA/cHAP composites were made by solubilising the polymer in chloroform solvent. After swirling PLA in chloroform at room temperature in a rotary evaporator, cHAP was added until PLA homogenisation was complete and ultrasonication was performed. After homogenising, the slurry was poured onto a refractory to create a film and dried in a fume hood for 24 h. After cutting into 3 × 3 mm pieces, the material was oven-dried at 50 °C for 48 h. A knife mill comminuted the dry material to 0.5 × 0.5 mm. This comminute material was extruded using a single-screw extruder to make a 1.75 mm filament wire. Finally, filament on a 3D printer was used to create mechanical analysis pieces in different forms. Table 2 compares PLA and cHAP mass to the volume of chloroform solvent used.

#### 2.2.2. Composite Filaments Production

The equipment used was a Noztek filament-making machine with a 1.75 mm output head, a purified water bath at 25 °C, and a wire roller. A low feed rate (4 g/min) was used to facilitate the dispersion of loads, resulting in residence time in the Noztek extruder of approximately 60 min. The rotation speed was fixed at 60 rpm. The extruder temperature was set at 175 °C in the matrix. The circular cross-section of the matrix used was 2 mm in diameter. The extruded materials were discharged on a conveyor with an air-cooling system, then cooled in the form of filament and later wound on a reel. Each extrusion lasted approximately 60 min and generated approximately 120 g of each material. The extruded materials obtained were vacuum dried for 12 h and then vacuum sealed for their characterisation. All processed materials are described in Table 2.

#### 2.2.3. Processing of Scaffolds by FDM

The equipment used to print the specimens was a commercial 3D printer, PRUSA i3. The prints were performed with a 0.4 mm nozzle and a 0.1 mm layer resolution. The platform was heated to 50 °C, and a spray adhesive was used as a fixer. All print parameters were controlled by Slicer software 4.0, installed on a computer connected to the 3D printer. The sample dimensions are 20 mm × 35 mm; the physical construction of the scaffolds was carried out on a PRUSA i3 FDM printer, consisting of a printing platform, with dimensions of 230 mm wide × 230 mm long × 200 mm high. The slicer software utilised the theoretical strand width and thickness to prepare the printer for printing the porous scaffolds, and the printer’s extrusion die had a diameter of 0.9 mm. PLA and composite filaments were printed at a nozzle speed of 30 mm/s. The initial layer of printing for PLA and PLA/cHAP was conducted at 190 °C (to achieve a solid adherence to the base plate), and subsequent layers were printed at 180 °C.

### 2.3. 3D-Printed Scaffold Characterisation

#### 2.3.1. Microscopic Observation

Microscopic examination can establish if the printed pieces’ mesostructured is acceptable and whether additives make material deposition problematic. A JSM-6510/JEOL SEM studied the samples’ microstructure. Solid samples were frozen in liquid nitrogen for 30 min and cryo-fractured using two metal tweezers. The samples were then carbon-taped to an aluminium support and gold-covered. A voltage of 5 kV and resolutions of 300× and 2000× were used. Particle sizes were assessed using commercially available software Image J. 1.54f. However, optical microscope photos were utilised to measure pore size in distinct groups of components. Pore morphology affects mechanical and biological qualities, with osseointegration most effective for pore diameters between 150–500 μm. Zhang et al. [27] examined how average pore size affects meniscus regeneration ability utilising in vitro and in vivo models. This study measured pore size using a scan electron microscope. Since only five pieces could be directly viewed and measured by optical microscopy, they were measured on the layer and the layer below it (Figure 5). Thus, 37 measurements were taken to calculate the average pore size of each structure.

#### 2.3.2. Thermal Characterisation

Thermogravimetric analysis (TGA) was performed using a computerised thermogravimetric analyser, and the thermal stability and degradation of nanofillers and composites were measured. TGA analysis was conducted at a heating rate of 10 °C/min, from approximately 50 °C to 800 °C, in an unsealed platinum crucible and synthetic air atmosphere at a flow rate of 60 mL min^−1^. The equipment used was the TGA Q500, TA Instruments. The mass of the analysed samples varied between 5 and 10 mg samples (powders with dimensions from 0.5 to 1 mm). Derived thermogravimetry was used to identify the maximum degradation temperature (peak temperature of the TGA curve). Differential Scanning Calorimetry (DSC) was performed using a computerised differential scanning calorimeter, and the composites’ crystallisation and melting temperatures and their corresponding heat fluxes were measured. The DSC analyses were carried out following three stages; specifically, the first heating was from 30° to 220 °C with a rate of 10 °C/min, followed by sudden cooling to 10 °C, keeping this temperature for 5 min, and finally, heating again up to 220 °C at a rate of 10 °C/min. The experiment was conducted in a nitrogen atmosphere at a 50 mL/min flow rate in a sealed aluminium crucible. The samples (powders with dimensions from 0.5 to 1 mm) were weighed between 5 and 10 mg and placed directly on the DSC.

#### 2.3.3. Mechanical Tests

Compression experiments were used to analyse the mechanical characteristics of 3D-printed scaffolds made from PLA and PLA/cHAP. A universal testing machine (Zwick Roell Z0.5 testing machine) with a 50 kN load cell was used to conduct the experiments. For 48 h before testing, all specimens were conditioned at 23 °C and 50% relative humidity, with a grip velocity in compression of 1 mm/min. The stiffness (E) of PLA and PLA/cHAP scaffolds was determined by determining the slope of the linear stress–strain relationship at low strains.

The tensile tests were performed using a set of five samples of each material (virgin PLA and PLA/cHAP composites) with nominal dimensions of 70 × 10 mm, using the EMIC DL3000 equipment with a 50kN load cell. The head speed was set constant at 5 mm/min. The ASTM D638-14 [32] standard was used for the tensile test specimens, which states that the ideal format for carrying out the test is the specimen with a rectangular section.

### 2.4. Machine Learning Implementation

#### 2.4.1. Data Description

The machine learning algorithm was developed using data from tensile and compression experiments conducted on PLA, PLA/cHAP 7.5%, and PLA/cHAP 10% samples. Each sample case comprised a total of 10 datasets each for optimised and unoptimised conditions, respectively. This data, focused on mechanical parameters, is integral for adequately characterising material properties, offering the potential for optimising structural designs in engineering applications [9,14,33]. Furthermore, the mechanical tests revealed that the in-plane mechanical performance exhibited variations with adding calcium hydroxyapatite. This variability presents an opportunity to train a predictive machine learning (ML) model.

#### 2.4.2. Data Preprocessing

Data preprocessing was conducted in two stages: label encoding and data normalisation. Encoding was necessary for the various PLA, PLA/cHAP 7.5%, and PLA/cHAP 10% samples to handle categorical data (i.e., variables that take on a limited, fixed number of values). Since most ML algorithms require numerical data, label encoding ensures each unique category is assigned a unique numerical label, which is fed into the ML algorithms more efficiently and allows these algorithms to process the data. The data were normalised by rescaling the features to a defined range, where any potential feature dominance is prevented and the impact of outliers mitigated, thereby improving the convergence and compatibility of the ML algorithm. The dataset features were described as follows: $X={{[X}_{1},\;\ldots ,\;X_{n}]}^{t}\;\in \;R^{nxm},$ where n and m represent the total number of observations and features transformed within a range of 0 to 1. This transformation was performed using:

(1) $$X_{N}=\frac{X-X_{min}}{X_{max}-X_{min}}$$

where $X_{N}$ is the normalised feature value, while $X_{max}$ and ${\;X}_{min}$ denote the maximum and minimum values of the selected feature, respectively. In this work, other preprocessing steps were avoided, and the experiments were repeated five times to ensure repeatability. Importantly, no other preprocessing techniques were applied to the data to ensure that underlying patterns in the data distributions were captured without influencing outliers. Consequently, the approach adopted in this study is to develop a robust predictive model in the presence of outliers.

#### 2.4.3. ML Regressor Algorithm Selection

The Lazy Predict library, a Python tool designed for rapid prototyping and evaluation, was utilised to streamline the training and assessment of multiple machine-learning models. It automatically applies default configurations and hyperparameters for efficient analysis. This tool enabled the mechanical prediction of the samples, deploying a pool of forty-two ML regression algorithms. Subsequently, a majority voting mechanism was executed to identify and retain models that achieved a minimum of 70% accuracy in forecasting the target variables pertinent to the materials studied. This study employed the Friedman statistic, a non-parametric statistical test, to ensure that the top-performing algorithms were not merely due to chance. The Friedman test was adopted in this study due to its robustness towards data normality [34]. This statistic is expressed as:

(2) $$X^{2}=\frac{12}{N(k+1)}\left(\sum R_{j}^{2}-\frac{{k(k+1)}^{2}}{4}\right)$$

where N is the number of items, k is the number of treatments, and $R^{2}$ is the sum of ranks for the $j^{th}$ Treatment. To determine the *p*-value, the computed Friedman statistic is compared to the chi-square distribution. The *p*-value, derived from the Friedman statistic, is compared to a significance level of 0.05. If the *p*-value is lower, the null hypothesis is rejected, confirming the superior performance of the regressor beyond chance. This criterion ensures that the focus is on the most promising models. Model predicting algorithms include ensemble learners (Adaptive Boosting, Extreme Gradient Boosting, and Random Forest regressors), support vector, and k-nearest neighbour regressors. Two of the regressors are briefly described below.

#### 2.4.4. Extreme Gradient Boosting Regressor

**Figure 2:**
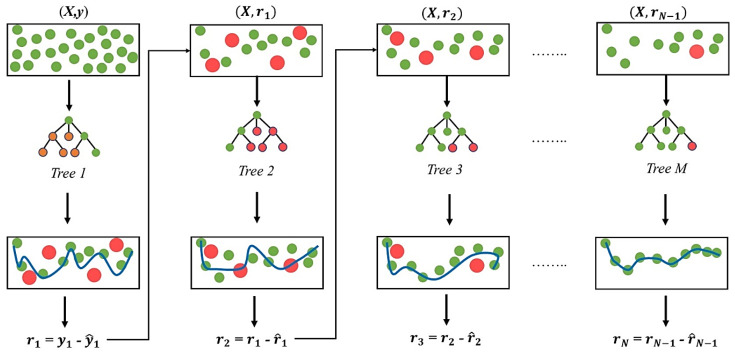
An illustration of regression using XGBoost.

The Extreme Gradient Boosting (XGBoost) regressor is an extension of the gradient boosting method, which functions by combining the predictions from multiple weak learners (i.e., individual decision trees) to create a strong learner. This is achieved by fitting a new tree to the residuals (the difference between the predicted and actual values) of the previous prediction, as illustrated in Figure 2. XGBoost supports different objective functions and evaluation metrics, making it adaptable to various regression problems [35]. It also includes an in-built regularisation technique to prevent overfitting and has several hyperparameters for model tuning.

#### 2.4.5. K-Nearest Neighbour Regressor

The k-nearest neighbours (K-NN) regressor is a non-parametric algorithm that predicts continuous values of a target variable by computing the average or weighted average of the k-nearest neighbours in the dataset. It utilises the Euclidean distance (or other metrics) to measure the distance between the data point to be predicted and all other data points in the training set. Subsequently, it selects *k* data points with the smallest distances to the target data point. It should be noted that the predicted value for the target data point is the weighted average of the target values of its k-nearest neighbours [14,36].

#### 2.4.6. Data Resampling and Model Training

Given the dataset’s size in this study, bootstrapping integrated with cross-validation was employed. This approach comprehensively assesses the model’s performance and objectively estimates uncertainties. Bootstrapping, as a statistical technique, involves randomly selecting n samples from the original dataset with replacement. This maintains the size of the original dataset, regardless of the potential presence of duplicate instances [37]. This ensured that both the training process and subsequent model evaluation were conducted on a representative dataset, thereby enhancing the reliability of the machine learning models.

#### 2.4.7. Hyper-Parameter Tuning and Model Training

**Figure 3:**
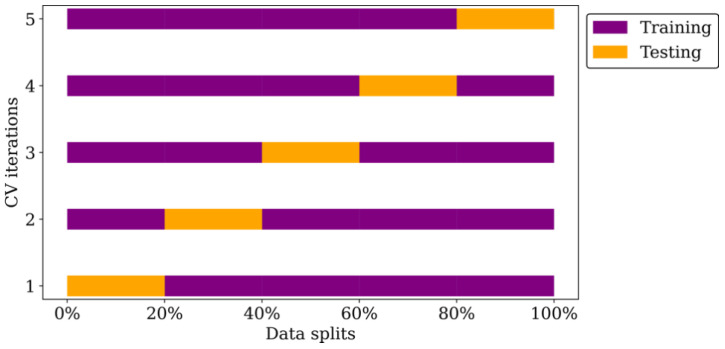
Illustration of cross-validation process for one hyperparameter combination.

The next step involves hyperparameter tuning, which is essential in determining the optimal set of hyperparameters for each machine learning algorithm. This process aims to enhance the model’s performance on unseen data. This study used grid search cross-validation (CV) for hyperparameter tuning. It creates a grid of all possible hyperparameter combinations and partitions the dataset into k = 5 folds. Each iteration trains the model on k-1 folds and evaluates it on the remaining fold, with rotations to ensure all folds are used for training and validation. Model performance for each hyperparameter combination is assessed by averaging the performance metric across all folds. Figure 3 illustrates this cross-validation process.

#### 2.4.8. Model Evaluation

MSE, MAE, and R-squared were used to determine the effectiveness and accuracy of the developed models. Previous studies have demonstrated the reliability of these metrics in assessing the performance of predictive models in regression analysis [38,39]. MSE represents the average squared difference between predicted and actual values of a model, while MAE signifies the average absolute difference between actual and predicted values. A closer value to 0 for MSE and MAE is desirable as it denotes better predictions of the exact material properties. Furthermore, R-squared quantifies the percentage of variation of y that *X* can explain in the regression model within a range between 0 and 1. A value of 1 indicates an excellent predictive performance, while 0 indicates a poor prediction. These metrics can be calculated as follows:

(3) $$MSE=\frac{1}{n}\sum_{i=1}^{n}{\left(y_{i}-{\hat{y}}_{i}\right)}^{2}$$

(4) $$MAE=\frac{1}{n}\sum_{i=1}^{n}\left|y_{i}-{\hat{y}}_{i}\right|$$

(5) $$R-squared=1-\left(\left(\sum_{i=1}^{n}{\left(y_{i}-{\hat{y}}_{i}\right)}^{2}\right)/\left(\sum_{i=1}^{n}{\left(y_{i}-\bar{y}\right)}^{2}\right)\right)$$

where $y_{i}\;and\;{\hat{y}}_{i}$ are the true and corresponding predicted values of the response variable Y for the *i*th case, respectively.

## 3. Results and Discussion

### 3.1. Visual and Dimensional Aspects of the Filaments and Scaffolds of PLA/cHAP

**Figure 4:**
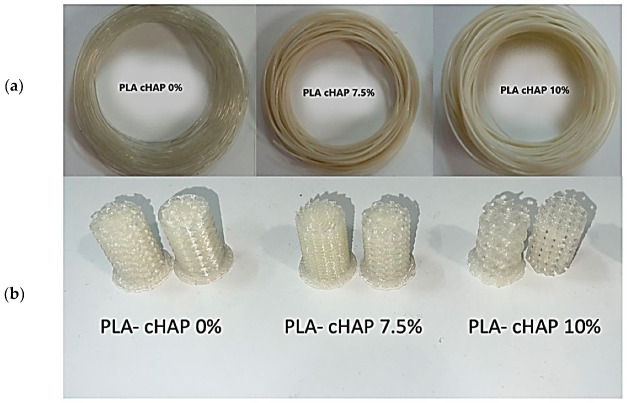
(**a**) Sample filaments obtained by extrusion, (**b**) stereoscopic micrographs of 3D printing scaffolds.

PLA/cHAP filaments were produced according to the methods described in Section 3.3 and are shown in Figure 4. These images provide visual insights into the produced filaments’ physical characteristics and structural variations. By examining these images, several valuable pieces of information can be obtained.

Visual Confirmation of Homogeneity: The pictures allow for a visual assessment of the degree of homogeneity achieved within the filaments at different concentrations. The presence of any visible agglomerates or inconsistencies in the filament structure can be readily identified.

Comparative Analysis: Comparing the pictures of filaments with varying concentrations of cHAP (0%, 7.5%, and 10%) enables a direct visual comparison of how adding cHAP affects the overall filament morphology. This visual comparison aids in understanding the impact of varying concentrations on the filament’s structural integrity. 

Particle Dispersion: By closely examining the images, one can assess the dispersion and distribution of cHAP particles within the PLA matrix. This insight is valuable in determining the effectiveness of the dispersion method used in the composite preparation process. Figure 4 below shows images of the PLA/cHAP scaffolds PLA/cHAP 0%, PLA/cHAP 7.5%, and PLA/cHAP 10%, manufactured according to the methods described in Section 3.4.

All PLA/cHAP filaments (PLA/cHAP 0%, PLA/cHAP 7.5%, and PLA/cHAP 10%) were produced with diameters of 1.75 mm ± 0.5 consisting of a diameter within the printing tolerance. PLA filaments with no calcium hydroxyapatite (PLA/0%cHAP) had a transparent and smooth texture, whereas PLA filaments with calcium hydroxyapatite (PLA/7.5% cHAP and PLA-/10% cHAP) had a whitish appearance and a rough texture, both of which were exacerbated by the increase in calcium hydroxyapatite content.

To address concerns about printability and potential nozzle clogging due to the addition of calcium hydroxyapatite (cHAP) nanoparticles, several strategies were implemented during the filament production and printing processes. Initially, minor clogging issues were observed, particularly with higher cHAP concentrations, likely due to nanoparticle agglomeration, a known issue when incorporating fillers into polymer matrices. For example, Zhou et al. [40] observed similar clogging problems in their study of nanoparticle-reinforced polymers and addressed this by optimizing extrusion parameters [40]. To mitigate this, the nozzle temperature was optimized, increasing it from 180 °C to 190 °C, as higher temperatures can prevent premature solidification of additives, an approach also recommended by Wonza et al. in their work with reinforced filaments [41]. Additionally, the extrusion speed was reduced to 30 mm/s, based on findings from other studies suggesting that slower speeds improve flow control and reduce the risk of nozzle blockages [42]. Ultrasonication was employed during filament preparation to ensure uniform dispersion of the cHAP particles, as demonstrated by Furukawa et al., who used ultrasonication to successfully break down nanoparticle agglomerates and enhance homogeneity [43]. These optimizations, supported by existing research, contributed to smooth extrusion, reliable print quality, and consistent layer adhesion

According to Ravi P et al. [11,44], scaffolds made of gyroid and Schwarz primitive lattices with rough surfaces provide the anchorage of cells and facilitate their deposition, adhering better to tissues. In contrast, scaffolds with very smooth surfaces, like those made from square lattices, are subject to micro-movements when used to produce scaffolds for maxi facial implants, which may cause damage to healthy tissue.

### 3.2. Microstructural Characterisation

**Figure 5:**
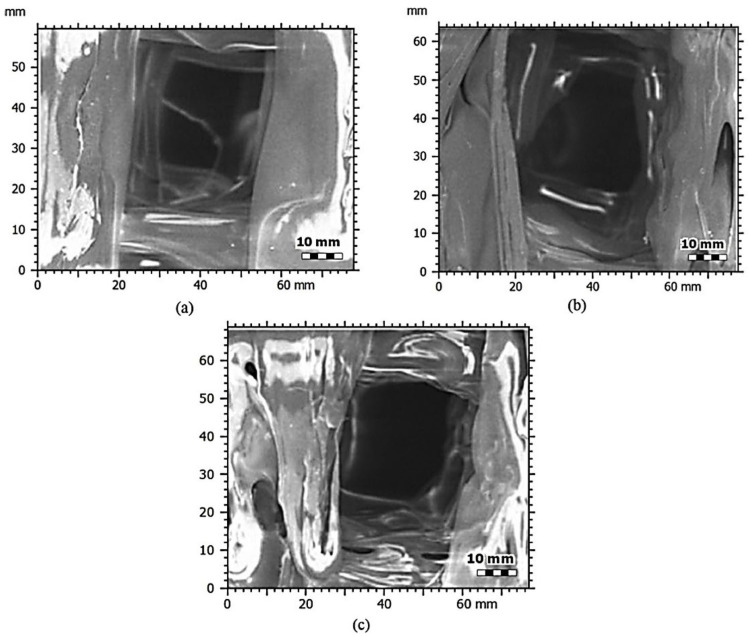
SEM image of the PLA/cHAP scaffold framework pores at (**a**) 100/0%, (**b**) 95/7.5%, and (**c**) 90/10% ratios.

The SEM images provided in Figure 5a–c showcase the internal architecture of the PLA, PLA/cHAP 7.5%, and PLA/cHAP 10% scaffolds, offering insights into how the incorporation of calcium hydroxyapatite (cHAP) at varying concentrations affects the structural characteristics of these scaffolds. The micrographs reveal significant differences in the pore morphology and distribution across the three composite types, which are crucial factors in determining the scaffolds’ potential for bone tissue engineering.

In Figure 5a, the pure PLA scaffold exhibits a relatively uniform but less defined pore structure than the composites containing cHAP. The pore walls appear smooth, indicating that while the PLA scaffold may provide a basic framework for cell attachment and nutrient flow, its mechanical strength and bioactivity might be limited. The lack of reinforcement in pure PLA often leads to reduced mechanical properties, as previous studies showed that pure PLA scaffolds struggled to maintain their structural integrity under physiological conditions, particularly in load-bearing applications [40,45].

In contrast, Figure 5b, representing the PLA/cHAP 7.5% scaffold, shows a more pronounced and consistent pore architecture. The introduction of 7.5% cHAP into the PLA matrix enhances the definition of the pores, contributing to a more robust internal structure. The cHAP particles likely act as nucleation sites during the printing process, which promotes a more uniform distribution of the PLA around the pores. This improved architecture is advantageous for bone tissue engineering, as it suggests that the scaffold can better support cell proliferation and differentiation due to the enhanced mechanical properties imparted by the cHAP. The literature supports this observation, indicating that scaffolds reinforced with hydroxyapatite generally exhibit increased mechanical stiffness and bioactivity, making them more suitable for supporting bone regeneration [46,47].

Figure 5c, which illustrates the PLA/cHAP 10% scaffold, presents the most distinct and well-defined internal architecture among the three. The higher concentration of cHAP further enhances the pore structure, providing a scaffold with superior mechanical properties and a more interconnected porous network. The pore walls are thicker and more rigid, which suggests that this scaffold would offer the best combination of mechanical strength and biological functionality. However, it is essential to note that while the increased cHAP content improves the structural integrity and bioactivity, it could also lead to increased brittleness of the scaffold, a common trade-off observed in composite materials. This balance between strength and brittleness must be carefully managed to ensure the scaffold remains functional under physiological conditions.

When comparing these observations with the literature, it is evident that adding cHAP significantly enhances the performance of PLA scaffolds. Studies by Zhang et al. [29] and Lu et al. [48] have similarly demonstrated that incorporating hydroxyapatite into polymer matrices improves mechanical properties and bioactivity, crucial for successful bone integration. The PLA/cHAP 10% scaffold, in particular, aligns with these findings, showing the most potential for clinical application in bone tissue engineering due to its optimal pore structure and mechanical characteristics.

In summary, the SEM analysis of the PLA, PLA/cHAP 7.5%, and PLA/cHAP 10% scaffolds highlights the significant impact of cHAP concentration on the scaffolds’ internal architecture and mechanical properties. While providing a basic structure, the pure PLA scaffold lacks the mechanical robustness and bioactivity necessary for adequate bone regeneration [49]. The PLA/cHAP 7.5% scaffold represents a marked improvement, offering a more defined pore structure and enhanced mechanical properties. With its superior internal architecture, the PLA/cHAP 10% scaffold presents the best option among the three, though considerations regarding its potential brittleness must be addressed. These findings are consistent with the existing literature, confirming the advantages of using cHAP-reinforced scaffolds in bone tissue engineering applications.

### 3.3. Thermal Stability

#### 3.3.1. Thermogravimetric Analysis

**Figure 6:**
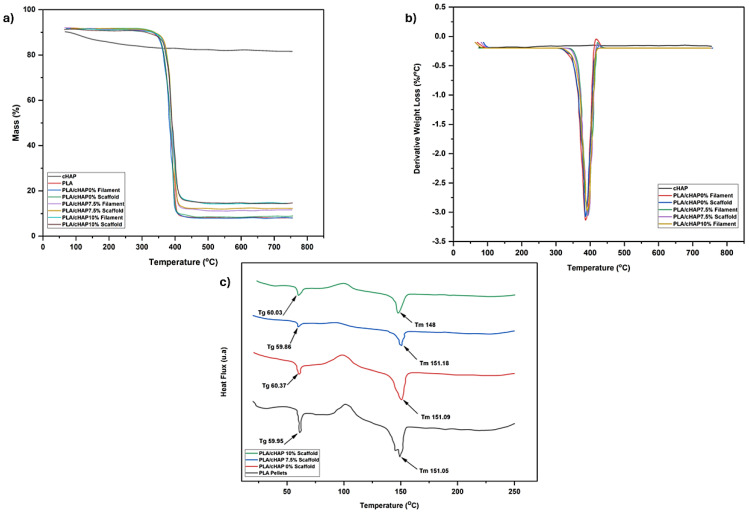
(**a**) General thermogram of the specimen, (**b**) general DTG curve of materials used in composites of the filaments and scaffolds obtained, (**c**) comparison of the DSC curves.

The thermogravimetric analysis revealed distinct stages of degradation for the materials, detailed in Figure 6 and Table 3. Initial degradation temperatures (T_i_) were marked by the onset of DTG peaks, indicating significant mass loss.

Maximum degradation temperatures $T_{max}were$ observed at the peak of the DTG curves, and final degradation temperatures ($T_{final}$) where the process stabilises are noted at the end of these peaks. Notably, calcium hydroxyapatite exhibited a single degradation stage, likely due to water loss, leaving a substantial residue at 800 °C, as per Zimina et al. [39]. The PLA and PLA/cHAP composites also degraded in a single step, with minimal change in the degradation temperature range. This suggests that scaffold production processes did not significantly alter PLA’s structure despite extensive thermomechanical treatments.

Interestingly, the PLA/cHAP 0% scaffold showed a noticeable decrease in initial degradation temperature compared to PLA alone, both as a pellet and after 3D printing, indicating less thermal stability. Conversely, scaffolds with 7.5% and 10% cHAP content increased initial degradation temperatures by 22.8 °C and 22.9 °C, respectively, suggesting enhanced thermal stability. This pattern aligns with findings from Pietrzykowska et al. [41], who noted similar improvements in thermal stability with cHAP in PLA scaffolds. Further, studies by Zhang et al. [27] and Oladapo et al. [29] demonstrated that adding 20% and 40% HA increased the initial degradation temperature of PLA scaffolds by 4 °C and 28 °C, respectively. The residue analysis also indicated an increase in residue quantities proportional to cHAP content in the composites.

#### 3.3.2. Differential Scanning Calorimetry

Table 4 details the thermal transitions and crystallinity degrees from Figure 6c’s graph. The second heating cycle was utilised to provide accurate and reliable thermal data. This cycle helps stabilise materials, eliminate initial conditioning effects, and establish a baseline to correct for instrument-related drift or noise. It is particularly critical for crystalline materials like PLA, revealing details about crystallisation and melting behaviours, thus enhancing the understanding of the material’s thermal properties [24,38]. This section discusses fundamental thermal properties, including the glass transition temperature ($T_{g}$), melting temperature ($T_{m}$), crystallisation temperature ($T_{c}$), and enthalpy of fusion (${∆H}_{f}$), which indicates the energy required for melting and enthalpy of crystallisation (${∆H}_{c}$), representing the energy released during crystallisation. The degree of crystallinity ($X_{c}$) measures the proportion of crystalline material. The DSC plots highlight PLA’s low crystallinity, showing a predominantly amorphous state similar to the findings by Zhang et al. [27].

In contrast, PLA/cHAP composites, particularly with 10% cHAP, show significantly higher crystallinity. This increase in Xc and an 8 °C reduction in T_c_ suggests better polymer-filler interaction and dispersion within the matrix, enhancing the crystalline properties as noted by Zhou et al. [40] and supported by Pietrzykowska et al. [40] in their research on PLA/HAP composites. Except for PLA pellets, which exhibited an amorphous behaviour, all materials demonstrated semicrystalline properties with characteristic changes at the glass transition temperature, exothermic crystallisation peak, and crystalline melting endothermic peak related to the melting of the crystalline phase of the material.

### 3.4. Mechanical Properties

**Figure 7:**
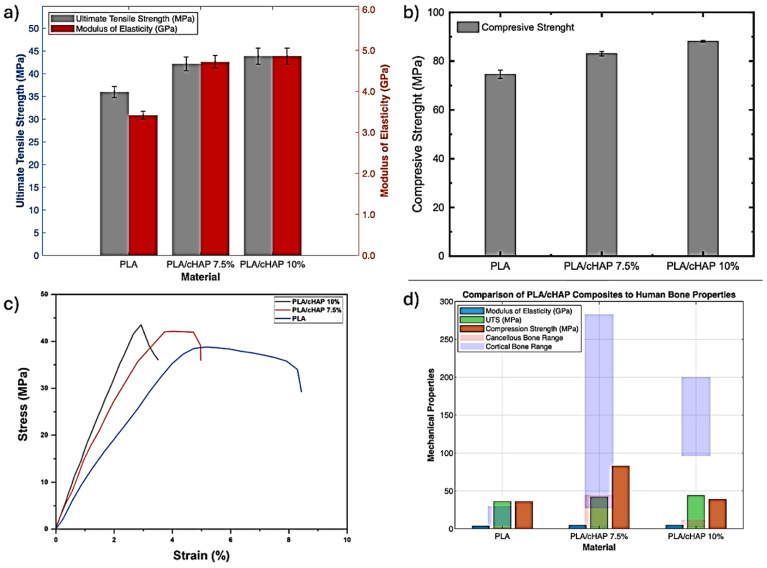
PLA/cHAP mechanical properties. (**a**) Tensile strength and elastic modulus, (**b**) compressive strength, (**c**) stress–strain plot, (**d**) comparison of PLA/cHAP composites to human bone properties.

Tensile characteristics of the produced PLA/cHAP composites are shown in Figure 7 and compared to those of pure PLA. It was first seen that the composites’ tensile strength and modulus were enhanced. The tensile strength and modulus of the modified PLA rose by 17.9% and 9.6% when cHAP was added at a concentration of 7.5%. The tensile strength and modulus increased by 20.1 and 30.6% when the cHAP loading was raised to 10% from plain PLA. In most cases, the enhanced tensile characteristics of PLA/cHAP composites at lower cHAP loadings (7.5%) may be attributed to the cHAP being evenly spread in the PLA matrix and so reinforcing the PLA matrix. Tensile characteristics may be improved due to dispersion and interfacial adhesion between the PLA matrix and cHAP.

For this reason, the increase in tensile modulus and strength may be partly attributed to the weak van der Waals forces created between the PLA matrix and cHAP [26,27]. Stress transmission between the cHAP and the PLA matrix increases the material’s tensile strength, even though there are some agglomerations of cHAP, as shown in the SEM micrographs (at more significant cHAP loading) [28]. Solution mixing may be the best method for creating composites with a high concentration of nanofiller [29], which may explain the lack of deteriorating effects on the tensile properties of the composites due to the aggregation of the cHAP at higher loading.

As seen in Table 5, the findings obtained for PLA/cHAP (7.5% & 10%) composites can match the tensile strength of cortical bones (50–60 MPa) [30], which is notable given that the tensile strength improved with an increase in cHAP loading. The PLA/cHAP composites nevertheless have tensile strengths and moduli higher than the range of cancellous bones (0.1–30 MPa and 20–500 MPa, respectively) [31]. Figure 7b shows the compressive strength of the PLA/cHAP composites prepared compared to neat PLA. Similarly, for tensile properties, the compressive strength of the PLA/cHAP composites displayed a general increase upon increased cHAP loading. For instance, with only 7.5% cHAP, the compressive strength of neat PLA increased by 10.1%. Optimum compressive strength was obtained upon the addition of 10% cHAP, which was 17.4% and 8.2% higher than the neat PLA and PLA/cHAP 7.5% composites, respectively; similar results were reported by Zare et al. [50] in their research. The continuous increase in the compressive strength of the PLA composites observed as the cHAP loading increased suggests the PLA matrix became more brittle upon the addition of cHAP [51]. Generally, incorporating nanofillers into a polymer matrix reduces the mobility of the polymer chain segments, hence inducing lower compressive strength. However, the non-formation of agglomerates causes the stress to be concentrated throughout the composites, thus increasing the compressive strength [52] while also causing the tensile strength to increase, as reported earlier upon higher cHAP loading.

### 3.5. Machine Learning Analysis

#### 3.5.1. Setup

The ML framework in this study heavily relied on Python, leveraging essential libraries like pandas, NumPy, scikit-learn, and matplotlib. These libraries played critical roles in performing numerical computations, preprocessing data, and developing machine learning models. The experimental data were imported and organised using the pandas’ data frame, with each PLA sample labelled for easy reference during numerical computations. Next, the variables underwent scaling to a range from 0 to 1. Depending on the scenario, the predictor variables included the specimen type, modulus, and compression or tensile mechanical properties. The compression or tensile mechanical property was designated as the response variable, with compression being exempted as a predictor variable when it served as the response variable. Following data preprocessing, the Lazy Predict algorithm evaluated model performance across forty-two ML regressors using their default hyperparameter settings. For each prediction of material properties, the dataset underwent reshuffling based on the NumPy random state generator, ensuring robustness in model assessment. The Lazy Predict algorithm was then employed for preliminary model evaluation. Subsequently, the Friedman statistic was utilised to determine the significance level among the ten results generated by Lazy Predict. The Lazy Predict model assessment outcome highlighted four top-performing regressors identified through majority voting across the material properties under consideration.

A linear regression algorithm was also incorporated as a benchmark to evaluate complex nonlinear patterns and feature interactions. In developing the prediction models, five regressor algorithms were utilised: AdaBoost, Random Forest, XGBoost, K-NN regressor, and Linear Regression. For regressor algorithms where hyperparameters significantly impacted model performance, grid search cross-validation was employed to identify optimal hyperparameter values that generalise well on the regressor algorithm. The grid search was configured with k = 5 CV folds, using MSE as the scoring parameter. Following this, data resampling and model training were carried out with 20 bootstrap instances, utilising resampling with replacement to maintain the original dataset size. Subsequently, the trained models were evaluated based on MSE, MAE, and R^2^ values. Moreover, visualisations depicting the actual versus predicted values were plotted to provide a more intuitive understanding of the model’s accuracy.

#### 3.5.2. Performance

The statistical analysis for all material properties predictions indicated a *p*-value > 0.05 after the second iteration. This suggests that the outcomes observed from the Lazy Predict algorithm and the majority voting system used in selecting the top-performing model were not merely due to chance. In Figure 6, a plot illustrates the Friedman statistic and *p*-value results for tensile prediction based on ten iterations. The Friedman statistic was calculated as 69.83 at the second iteration, and the corresponding *p*-value was 0.0024. These results provide statistical evidence supporting the effectiveness and reliability of the model selection process. After the model selection process, the chosen model was trained using the training data, and predictions were generated for both the training and testing datasets. Subsequently, the model’s performance was assessed using MSE, MAE, and R^2^. Additionally, plots were generated to visualise the relationship between each model’s actual and predicted values. These evaluations provide insights into the model’s ability to generalise and accurately predict the target material properties.

#### 3.5.3. Evaluation Results

Ensemble learners such as AdaBoost, Random Forest, and XGBoost, along with K-NN regressor, in the selected regressor algorithms showcased their capability to capture nonlinear relationships and interactions between variables. Furthermore, these algorithms exhibited robustness in handling outliers, particularly for unoptimised specimen samples. An additional advantage of these algorithms is their ability to operate without making assumptions about the underlying data distribution. This attribute is particularly advantageous for datasets whose distribution may not be easily modelled. The relatively low performance of the linear regression model can likely be attributed to its inherent limitation in dealing with nonlinear patterns and relationships. This highlights the importance of considering more flexible, nonlinear models when dealing with multidimensional and intricate relationships among features. Table 6 presents the model evaluation results for compression and tensile material property prediction, providing a comprehensive overview of the performance of each model in predicting these properties.

**Figure 8:**
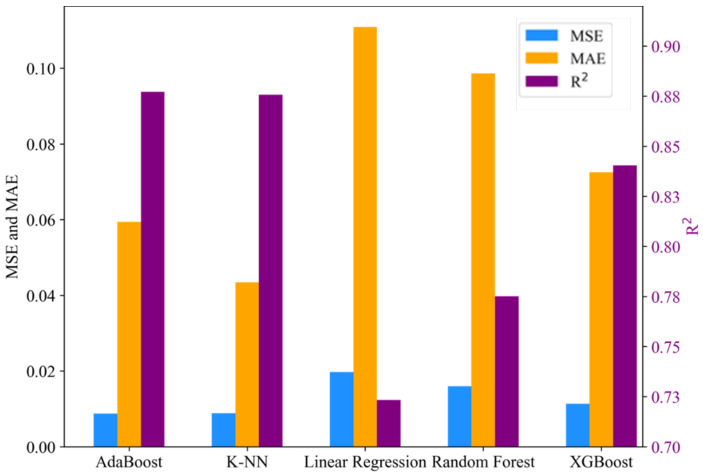
Model performance result for compression material property prediction.

**Figure 9:**
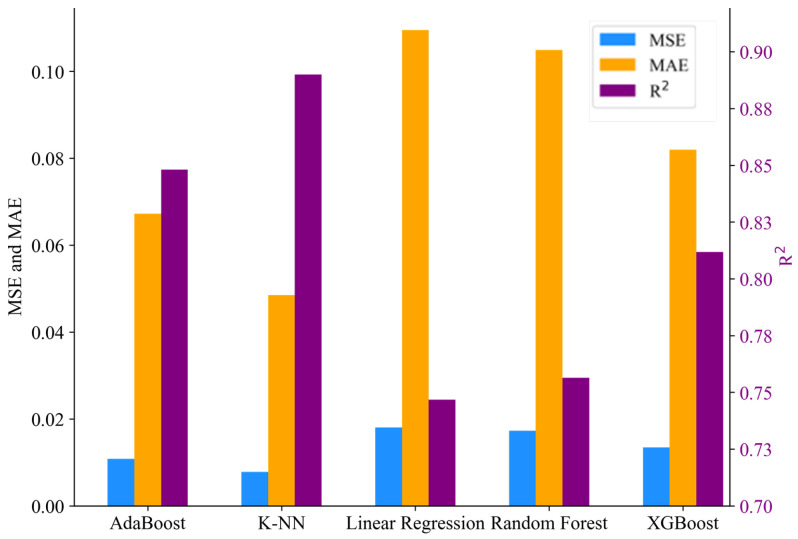
Model performance result for tensile material property prediction.

The models demonstrating the highest prediction performance, as indicated by the MSE, MAE, and R2 values, are highlighted in bold font. Excluding the linear regression model, included as a benchmark, all models exhibited predictive performance ranging between 82% and 92% for compression material property prediction. In the case of tensile material property prediction, performance ranged between 84% and 88% for all regressors except for random forest and linear regression. Due to the sensitivity of MSE to outliers, it yielded better performance results than the MAE values for all material properties considered. This comparison is illustrated in Figure 8 and Figure 9 which depict bar charts for compression and tensile prediction models, respectively.

**Figure 10:**
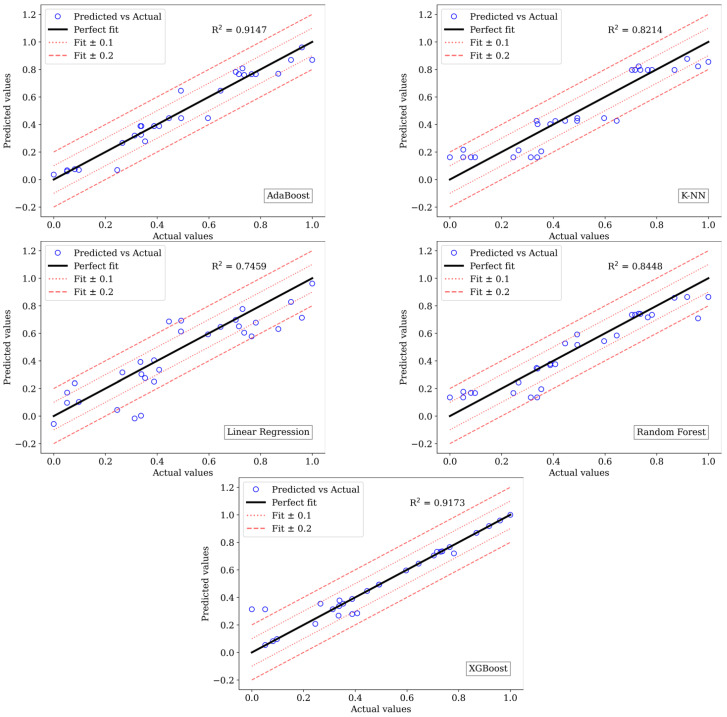
Predicted versus actual values of models for compressive strength.

**Figure 11:**
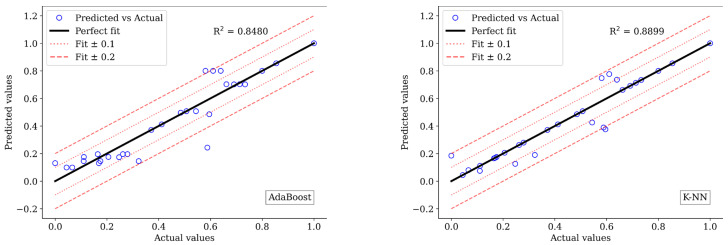

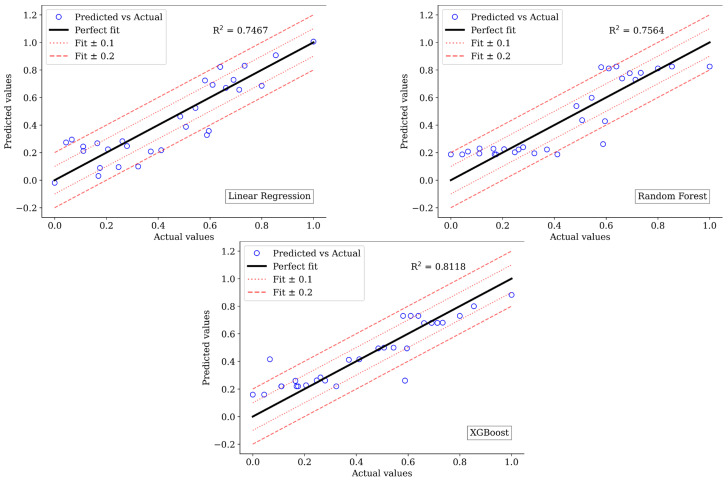
Predicted versus actual values of models for tensile strength.

Specifically, the models constructed using XGBoost and AdaBoost demonstrated optimal performance in predicting compression and tensile properties, respectively, for PLA composites. For predicting compressive strength, the XGBoost model achieved peak performances with MSE, MAE, and R^2^ values of 0.0067, 0.0384, and 0.9173, respectively. Regarding tensile strength prediction, the XGBoost model yielded MSE, MAE, and R^2^ values of 0.0087, 0.0594, and 0.8772, respectively. Graphical representations of the predicted versus actual compressive and tensile strength values are provided in Figure 10 and Figure 11 respectively.

The presented plots illustrate the relationship between the actual and predicted values relative to the ideal fit line. Notably, for both compressive and tensile strength of PLA, the actual and predicted values based on XGBoost are closely aligned with the perfect fit line compared to other models. Additional lines have been incorporated into the plots to visualise deviations from the predictions. The dotted lines represent a range of ± 0.1 deviations from the actual values, providing a reasonable approximation. On the other hand, dashed lines indicate a ±0.2 deviation from the actual values, representing a slightly more significant but still acceptable deviation. Points falling outside this ±0.2 range denote significant prediction errors. These visual aids help assess the prediction models’ accuracy and reliability. Among the models evaluated, only the AdaBoost model for predicting both compressive and tensile material properties exhibited values within the ±0.2 range, as observed in Figure 10 and Figure 11. In these figures, it is evident that linear regression falsely predicted several actual values at various points.

Additionally, false predictions within the range of ±0.02 were noticeable for most models, aside from the AdaBoost models. Consequently, the models can guarantee forecasts with up to 99% accuracy outside the ±0.2 range area for both tensile and compressive material property prediction for optimised and unoptimized PLA under various cHAP inclusion volumes. This suggests that while the models generally perform well, there are instances where they may deviate from accurate predictions, particularly within a narrow margin. This strong correlation between the model predictions and the experimental data underscores the effectiveness of machine learning models, particularly XGBoost and AdaBoost, in predicting the mechanical properties of PLA/cHAP composites. These predictions are crucial for understanding the inherent properties of the materials, but also play a significant role in practical applications. For instance, knowing these composites’ exact tensile and compressive strengths allows for more accurate and tailored applications in biomedical engineering, particularly in bone tissue engineering, where the mechanical demands are stringent. The predictive power of these models also facilitates the optimisation of the composite materials during the design phase, allowing engineers to simulate various formulations and their outcomes without the need for extensive physical testing. This capability significantly reduces the time and cost of developing new materials and enhances the ability to innovate more rapidly and efficiently.

Moreover, the high degree of accuracy these models provide ensures that the final products are safe and effective for their intended use, ultimately leading to better patient outcomes in medical applications. Integrating machine learning techniques in characterising PLA/cHAP composites has proven to be a valuable tool in advancing material science. It has provided a robust platform for predicting and understanding the mechanical behaviours of composite materials under various conditions, which is paramount for their application in demanding environments like those encountered in medical implants and other structural applications. The continued refinement of these models and their application to a broader range of materials will likely yield even greater insights and innovations in the future.

## 4. Conclusions

This research successfully demonstrated the significant potential of 3D-printed PLA/cHAP composite scaffolds for applications in bone tissue engineering. Through meticulous experimentation, it was established that incorporating calcium hydroxyapatite (cHAP) into the PLA matrix markedly improves the mechanical properties, including tensile and compressive strength, as well as the thermal stability of the scaffolds. Among the composites studied, the PLA/cHAP 10% scaffold emerged as the most promising candidate, exhibiting superior mechanical performance that aligns with the stringent requirements for load-bearing applications in regenerative medicine.

The microstructural analysis, facilitated by SEM, provided valuable insights into the internal architecture of these scaffolds. Adding cHAP enhanced the scaffold’s pore structure, creating a more defined and interconnected porous network. This structural refinement is crucial as it mimics the trabecular architecture of natural bone, promoting cellular infiltration, nutrient transport, and, ultimately, osteointegration. The improved pore architecture, combined with the mechanical robustness conferred by the cHAP, underscores the suitability of these scaffolds for clinical applications in bone regeneration.

Furthermore, the integration of machine learning models, particularly ensemble techniques like XGBoost and AdaBoost, has proven to be a powerful tool in predicting the mechanical properties of the composites. This approach streamlines the material design process and significantly reduces the need for extensive and costly experimental trials. By accurately forecasting the behaviour of these scaffolds under various conditions, machine learning has paved the way for the development of optimised, patient-specific scaffolds with tailored mechanical and biological properties.

In conclusion, the findings of this study demonstrate that PLA/cHAP composite scaffolds hold considerable promise for advancing bone tissue engineering. The combination of enhanced mechanical properties, refined pore architecture, and the predictive power of machine learning makes these scaffolds solid contenders for future clinical applications. Integrating these composites into clinical practice could significantly improve outcomes in bone regeneration, particularly in cases requiring robust and biologically active scaffolds. Future work should focus on optimising the fabrication process, exploring the long-term biodegradability and biocompatibility of these composites in vivo, and expanding the use of machine learning models to other composite materials and scaffold designs.

## Figures and Tables

**Table 1:** Properties of the PLA used [31].

Property	Value	ASTM Method
Relative density (g/cm^3^).	1.24	D792
Stress (MPa)	36	D638
Poison ratio	2.5	
Elastic modulus (GPa)	3.5	
T_m_ °C	151.5	E2092
Tg °C	55.1	

**Table 2:** Weight ratio of PLA cHAP to the volume of chloroform used.

Composites	Mass of PLA (%)	Mass of cHAP (%)	Volume of Chloroform (l)
PLA/cHAP	100	0	4.5
PLA/cHAP	92.5	7.5	4.5
PLA/cHAP	90	10	4.5

**Table 3:** Thermal degradation data of materials analysed by TG-DTG.

Sample	Degradation Temperature (°C)			
	$T_{i}$	$T_{max}$	$T_{final}$	% Final Residue
PLA pellets	320.5	387	426.8	0
PLA/cHAP 0% filament	318	384.2	426.9	0.11
PLA/cHAP 7.5% filament	321.7	390.5	430.8	3.95
PLA/cHAP 10% filament	319	389	427.9	7.67
PLA/cHAP 0% scaffold	297.7	385.3	426.2	0.27
PLA/cHAP 7.5% scaffold	320.5	390.3	431.3	4.88
PLA/cHAP 10% scaffold	320.6	390.9	431	7.75
Calcium hydroxyapatite				90.33

**Table 4:** Thermal transitions of the materials analysed by DSC relative to the second heating cycle.

Sample	Second Heating Cycle					
	$T_{g}$	$T_{m}$	$T_{c}$	${∆H}_{f}$	${∆H}_{c}$	$X_{c}$
	(°C)	(°C)	(°C)	(J/g)	(J/g)	(%)
PLA Pellets	60.03	151.05	-	2.51		2.6
PLA/cHAP 0% filament	60.15	151.09	123.50	21.54	−17.20	23.14
PLA/cHAP 7.5% filament	59.62	151.24	127.00	14.22	−12.54	16.51
PLA/cHAP 10% filament	58.90	150.06	115.39	22.25	−22.75	26.55
PLA/cHAP 0% scaffold	60.37	150.64	122.60	22.40	−24.31	24.06
PLA/cHAP 7.5% scaffold	59.86	151.18	125.58	19.63	−19.52	26.39
PLA/cHAP 10% scaffold	59.95	148.00	114.79	25.11	−25.86	29.96

**Table 5:** Comparative data from mechanical tests with human bones.

Composites	Modulus of Elasticity (GPa)	UTS (MPa)	Compression Strength (MPa)
Human Cancellous Bones [53]	0.3–3	1.5–45	2–12
Human Cortical Bones [53]	4–30	27–283	96–200
PLA	3.42	36	35.9
PLA/cHAP 7.5%	4.72	42.16	83.01
PLA/cHAP 10%	4.86	43.88	38.81

**Table 6:** Model evaluation results for compression and tensile material property prediction.

Model	Compression			Tensile		
	MSE	MAE	R^2^	MSE	MAE	R^2^
AdaBoost	0.0069	0.0500	0.9147	0.0087	0.0594	0.8772
K-NN	0.0144	0.0964	0.8214	0.0088	0.0434	0.8757
Linear Regression	0.0205	0.1159	0.7459	0.0197	0.1109	0.7233
Random Forest	0.0125	0.0823	0.8448	0.0160	0.0986	0.7751
XGBoost	0.0067	0.0384	0.9173	0.0113	0.0725	0.8405

## Data Availability

The original contributions presented in the study are included in the article, further inquiries can be directed to the corresponding author.
